# Supplementary material for: MAGEA4 Coated Extracellular Vesicles Are Stable and Can Be Assembled In Vitro
Source: Int J Mol Sci. 2021 May 14;22(10):5208. doi: 10.3390/ijms22105208 (PMC8155938; doi:10.3390/ijms22105208)
Supplement: Supplementary file 1 [file ijms-22-05208-s001.zip › ijms-1194584-SUP-PROOF DONE.pdf]

SUPPLEMENTARY MATERIALS

MAGEA4 Coated Extracellular Vesicles are Stable and Can Be Assembled in Vitro

Olavi Reinsalu [1], Anneli Samel [1], Elen Niemeister [1], and Reet Kurg [1*]

1 Institute of Technology, University of Tartu, Tartu, 50411, Estonia; olavi.reinsalu@ut.ee; anneli.samel@ut.ee; elen.niemeister@gmail.com; reet.kurg@ut.ee

* Correspondence: reet.kurg@ut.ee; Mailing address: Institute of Technology, University of Tartu, Nooruse 1, 50411 Tartu, Estonia

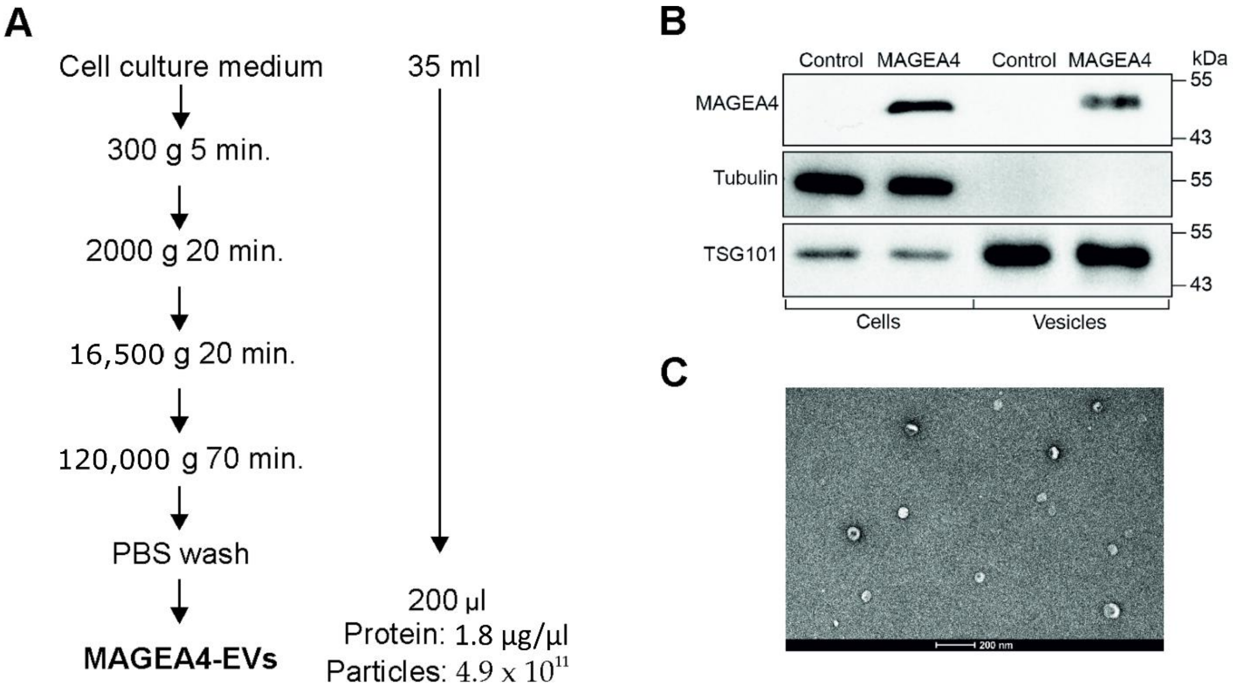

Figure S1. The characterization of MAGEA4 carrying extracellular vesicles (MAGEA4-EVs). (**A**) The scheme of MAGEA4-EV isolation and purification procedure. The vesicles were isolated from cell culture medium using differential centrifugation. After washing the pellet with PBS, the vesicles were ultracentrifuged and resuspended in PBS. (**B**) Western blot analysis of cells transfected with MAGEA4 expression plasmid and EVs isolated from the cell culture media of the cells. Cells transfected with an empty vector was used as control. (**C**) Transmission electron microscopy image of MAGEA4-EVs. The image was taken as described in [53].

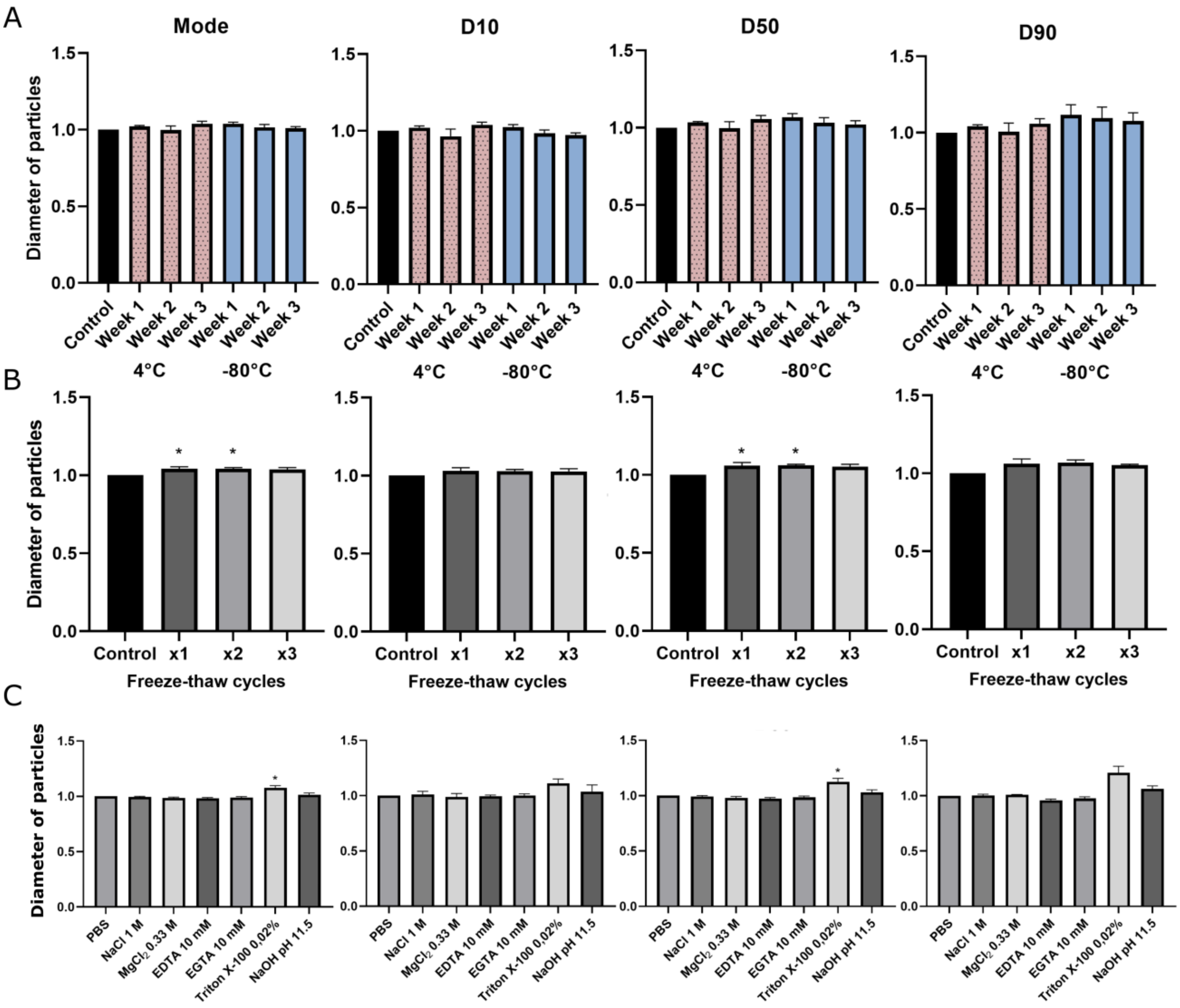

Figure S2. The particle size parameters of MAGEA4-EVs of different treatments. The normalized particle size distribution values as measured by NTA in following experiments: **(A)** MAGEA4-EVs were stored at 4 °C or -80 °C for up to 3 weeks; **(B)** MAGEA4-EVs were subjected up to three freeze-thaw cycles; **(C)** MAGEA4-EVs were treated with seven PBS based solutions: PBS as control, 1 M NaCl, 0.33 M $MgCl_2$, 10 mM EDTA, 10 mM EGTA, 0,02% Triton X-100 and 3.5 mM NaOH at pH 11.5. *- The difference of the sample value compared to control sample value is statistically significant with p-value < 0.05.

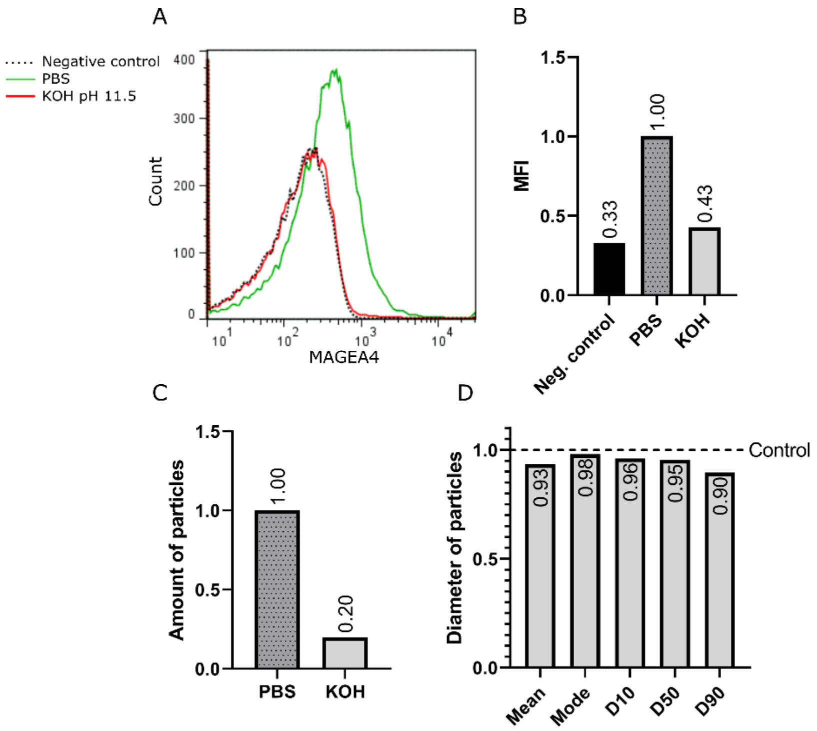

Figure S3. KOH pH 11.5 treatment is detrimental for MAGEA4-EVs. (A) A flow cytometry analysis of the MAGEA4 presence on the surface of the vesicles using anti-MAGEA4 and Alexa 488-conjugated secondary antibodies. The dotted line is a negative control (latex beads without EVs) and PBS marks the positive control (MAGEA4-EVs without any treatment). **(B)** The corresponding mean fluorescence intensity values (MFI) of the flow cytometry histograms in (A). **(C)** The amount of particles detected through NTA. **(D)** The size parameters of detected particles of KOH treated sample. The values in (B), (C) and (D) were normalized against corresponding PBS treated sample (positive control) values, which were always set as 1.
